# Reduce and Control: A Combinatorial Strategy for Achieving Sustained HIV Remissions in the Absence of Antiretroviral Therapy

**DOI:** 10.3390/v12020188

**Published:** 2020-02-08

**Authors:** Roland Schwarzer, Andrea Gramatica, Warner C. Greene

**Affiliations:** Gladstone Institute of Virology and Immunology, Departments of Medicine, Microbiology & Immunology, University of California, San Francisco, CA 94158, USA; roland.schwarzer@gladstone.ucsf.edu (R.S.); or ang4015@med.cornell.edu (A.G.)

**Keywords:** HIV, cure, block and lock, shock and kill, genome editing, reduce and control

## Abstract

Human immunodeficiency virus (HIV-1) indefinitely persists, despite effective antiretroviral therapy (ART), within a small pool of latently infected cells. These cells often display markers of immunologic memory and harbor both replication-competent and -incompetent proviruses at approximately a 1:100 ratio. Although complete HIV eradication is a highly desirable goal, this likely represents a bridge too far for our current and foreseeable technologies. A more tractable goal involves engineering a sustained viral remission in the absence of ART––a “functional cure.” In this setting, HIV remains detectable during remission, but the size of the reservoir is small and the residual virus is effectively controlled by an engineered immune response or other intervention. Biological precedence for such an approach is found in the post-treatment controllers (PTCs), a rare group of HIV-infected individuals who, following ART withdrawal, do not experience viral rebound. PTCs are characterized by a small reservoir, greatly reduced inflammation, and the presence of a poorly understood immune response that limits viral rebound. Our goal is to devise a safe and effective means for replicating durable post-treatment control on a global scale. This requires devising methods to reduce the size of the reservoir and to control replication of this residual virus. In the following sections, we will review many of the approaches and tools that likely will be important for implementing such a “reduce and control” strategy and for achieving a PTC-like sustained HIV remission in the absence of ART.

## 1. Introduction

The history of human immunodeficiency virus (HIV-1)/acquired immunodeficiency syndrome (AIDS) is a story of initial fear and panic followed by rapid, frankly stunning, scientific and medical progress [1]. Since the 1983–1984 discoveries of the human immunodeficiency virus (HIV-1) [2] and its etiologic linkage to the acquired immunodeficiency syndrome (AIDS) [3,4,5], this virus has been extensively dissected, its pathogenic mechanisms defined, and the host’s defensive immune responses well characterized. This deep understanding of the virus accelerated development of antiretroviral therapy (ART) principally targeting the major enzymes of the virus. Now, more than 30 different antiretroviral drugs are available. With access to and compliance with these drugs, viremia in essentially every HIV-infected individual can now be suppressed to undetectable levels. These therapeutic advances will be recorded as a true milestone in the history of modern medicine. 

However, despite this progress, we still do not have an effective prophylactic vaccine for the uninfected, or a safe and scalable cure for those already infected. Currently, over 37.9 million people are HIV-positive (UNAIDS 2019 report). Due in large part to the heroic efforts within programs like PEPFAR (https://www.hiv.gov/federal-response/pepfar-global-aids/pepfar) and the Global Fund (https://www.theglobalfund.org/en/), 23 million infected people are now receiving life-saving ART throughout the world including developing countries, where most new infections are occurring. However, the multi-billion-dollar annual investment by the developed countries required to fund daily ART in the developing world is becoming increasingly uncertain due to multiple factors, including AIDS fatigue among donors and changing international priorities. Those not currently being treated will undoubtedly prove the hardest to reach in future efforts. A safe, effective and scalable HIV cure would be medically and financially transformative for the world, and would certainly help sub-Saharan Africa bring a close to its long struggle against HIV/AIDS. 

### 1.1. HIV Latency and the Latent Reservoir

Following entry into CD4-expressing target cells, HIV-1 integrates into the host genome, establishing the HIV provirus. Two fates are possible for infectious proviruses: i) productive infection with spread of virions to new cellular targets or ii) establishment of latency characterized by little or no expression of viral proteins. Of note, most latent HIV proviruses are defective [6,7]. For every infectious HIV provirus there are at least 100, if not more, defectives present [8]. Although these defective viruses do not give rise to infectious progeny, they can produce inflammation through the intermittent production and release of viral RNA and proteins [7,9], including novel, unspliced RNA species as well as chimeric HIV proteins [10]. Increased inflammation favors further spread of infectious virus [11]. The relative lack of inflammation found in post-treatment controllers may play a special role in their ability to control spread of the virus. 

In blood, latent proviruses are primarily found in central, transitional, and effector memory CD4 T cells as well as in T-follicular helper (Tfh) cells and, to a lesser extent, in CD4 T stem memory cells (Tscm) [12,13] and hematopoietic stem and progenitor cells (HSPCs) [14]. The self-renewing properties of Tscm and HSPCs could play a key role in reservoir maintenance while lymphoid follicles, where Tfh cells primarily reside, could provide a site of relative immune privilege for infected reservoir cells due to reduced ingress of cytotoxic T lymphocytes (CTLs) [15,16,17].

With few exceptions [18,19,20], most studies of latency in HIV-infected individuals have involved the analysis of blood [21]. Going forward, it will be important to compare and contrast properties of HIV latency found in lymphoid tissue versus blood, but this will require better access to tissue [22]. Effector memory T cells may play an especially prominent role as cellular hosts for the latent reservoir in tissues [23].

Within the myriad of memory T cell subsets, multiple factors impact whether the virus is latent or not. These include the site of proviral DNA integration [24,25,26,27,28,29,30], the low abundance, or cytoplasmic sequestration, of key cellular transcription factors in resting cells [31], the presence of repressive epigenetic modifications [32,33,34,35], impaired RNA splicing [36] and decreased nuclear export, and the reduced translation of these viral RNAs [37]. One newly described mechanism through which HIV helps ensure its own latency involves the action of its antisense transcript (AST) generated within the 3’ LTR. AST binds to complementary sequences in the 5’ LTR and, like long non-coding RNAs, can promote recruitment of the Polycomb Repressor Complex 2 (PRC2) and induction of silencing through trimethylation of lysine 27 on the H3 histone (H3K27me3) [38]. 

### 1.2. Post-Treatment Control: A Blueprint for “Reduce and Control?”

Although almost all HIV-infected persons experience rapid viral rebound following ART interruption, rare individuals (<10%, [39,40]) exhibit sustained virologic suppression and persistently high CD4 T cell counts for months or years after treatment cessation. These “post-treatment controllers” (PTCs) [41,42,43] illustrate that it is possible to establish durable HIV control after infection. While the exact mechanism operating in PTCs is unclear, these individuals have a small reservoir [44], a low degree of inflammation, and relatively weak HIV-specific CTL activity [42,45]. Of note, recent interest has focused on the potential role of NK cells in PTC. For example, Pohlmeyer et al. have identified CD11b+, CD57-, CD161+, Siglec7+ subpopulations of CD56dim, CD16+ NK cells that are prominent in HIV controllers but not in HIV non-controllers [46].

Most of the approaches now in the clinic seek to achieve long-term control of a replication-competent reservoir (a “sustained viral remission”). PTCs appear to represent natural examples of the implementation of an effective “reduce and control” strategy [41]. We urgently require a better understanding of the molecular and immunological basis for post-treatment control. 

### 1.3. An Overview of HIV Cure Approaches

The first attempts to eliminate the latent reservoir focused on earlier and intensified use of ART. However, this approach has proven ineffective [47], in part because the reservoir is formed so quickly [48,49] and remains stable for so long [50,51]. Additionally, latent HIV proviruses are not adversely affected by ART. It became clear that novel therapeutic strategies were necessary to attack the latent reservoir. 

**Figure 1:**
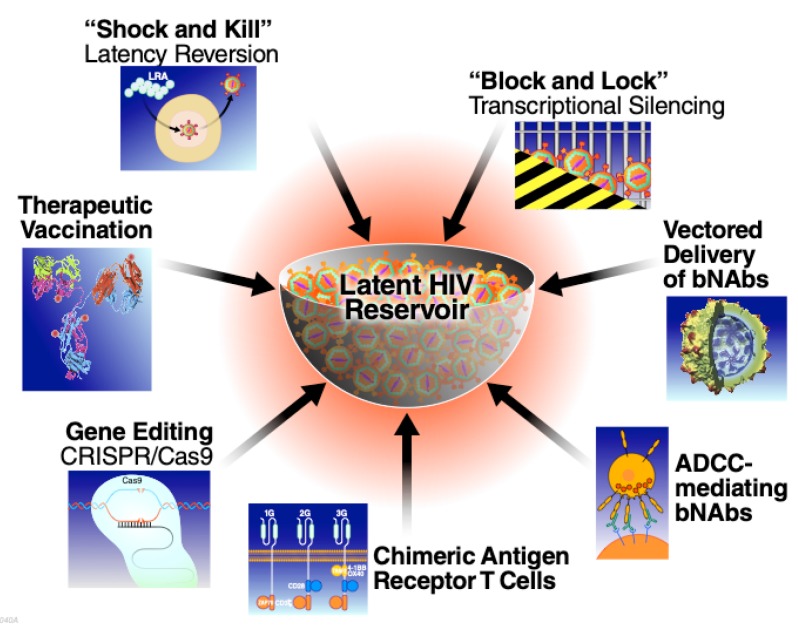
Therapeutic approaches being explored aimed at long term neutralization of the latent HIV reservoir. (bNAbs, broadly neutralizing HIV antibodies; ADCC, antibody dependent cellular cytotoxicity).

In the following sections, we provide an overview of many of the major HIV-1 cure strategies currently being pursued (Figure 1). First, we consider the existing immune-based approaches and then specifically focus on two complementary but not necessarily mutually exclusive strategies––“shock and kill” [52] and “block and lock” [53]. Both of these approaches could form important components of an overall “reduce and control” strategy aimed at recapitulating post-treatment control. Finally, we consider recent efforts to apply gene editing technologies to HIV cure efforts. 

### 1.4. Facilitating Immunological Control of HIV Infections

One example of an immune-based treatment for HIV infections is a therapeutic vaccine. In contrast to their preventive counterparts, therapeutic vaccines are not administered preemptively, but rather after infection has occurred. As such, a therapeutic vaccine must elicit a different or stronger type of immune response than typically occurs as a result of natural infection. A successful therapeutic vaccine could suppress viral replication and spread so effectively as to prevent viral rebound after discontinuation of ART [54]. Therapeutic HIV vaccines have had a generally disappointing record, likely in part due to the fact that early candidates did not induce sufficiently broad responses to control viral escape mutants. More promising results are now emerging in primate studies incorporating rigorous analytical treatment interruptions as a measure of vaccine effectiveness [55,56,57]. Combining an effective therapeutic vaccine with an agent(s) capable of reducing reservoir size could enable a successful “reduce and control” strategy. 

Another novel technology that seeks to facilitate immunological control of HIV-1 is the long-term expression of broadly neutralizing antibodies (bNAbs) delivered by viral vectors [58]. Multiple animal and human studies support the therapeutic potential of this approach, which would also render adherence issues moot. Nevertheless, long-term expression of bNAbs based on a vector-mediated gene transfer is, as technology, in its infancy. One concern with this method is the induction of anti-idiotypic immune responses against the humanized antibodies or immune responses to components of the viral vector [58]. Additionally, some classes of bNAbs recognizing glycan epitopes within Env have recently been shown to interact with uninfected cells, and thus could elicit unexpected and unwanted long-term toxicity [59].

bNAbs also can induce strong antibody-dependent cell cytotoxicity (ADCC) through interactions with FcRγ on natural killer cells. Clinical data show that the presence of antibodies that trigger ADCC is correlated with slower disease progression and reduced mortality [60]. Moreover, non-neutralizing antibodies mediating ADCC were identified as a correlate of protection in the RV144 HIV vaccine trial [61]. On the therapeutic front, passive administration of two bNAbs soon after infection of rhesus macaques with simian/human immunodeficiency virus (SHIV) was found to produce a potent CD8 T cell immune response that in 4 of 13 animals suppressed viremia for more than 2 years [62]. However, an open-label clinical trial involving administration of the VRC01 bNAb did not lead to persistent viral suppression following treatment interruption, although a delay in the time to viral rebound was observed [63]. Additional studies are required to more fully define the biological effects of bNAb administration in vivo. 

Recently, chimeric antigen receptor T cells (CAR-T cells) received FDA approval for the treatment of acute lymphoblastic leukemia and non-Hodgkin’s lymphoma [64,65]. Several studies, underway or recently published [66,67,68], are exploring CAR-T cells as a strategy to target HIV-1-infected cells. This technology is also in its infancy but holds great promise as a means to clear virus-infected cells, with minimal or no off-target effects. Due to their high specificity and long survival, CAR-T cells could in theory curb viral replication and spread in infected individuals, thus enabling both a reduction in reservoir size and the control of residual virus production. However, this approach is currently too expensive for broad-scale use. One the other hand, the CAR-T strategy will become more practical, markedly less expensive, and potentially scalable when universal allogeneic donor cells become available. 

## 2. In Vitro and In Vivo Reactivation of Latent HIV

**Figure 2:**
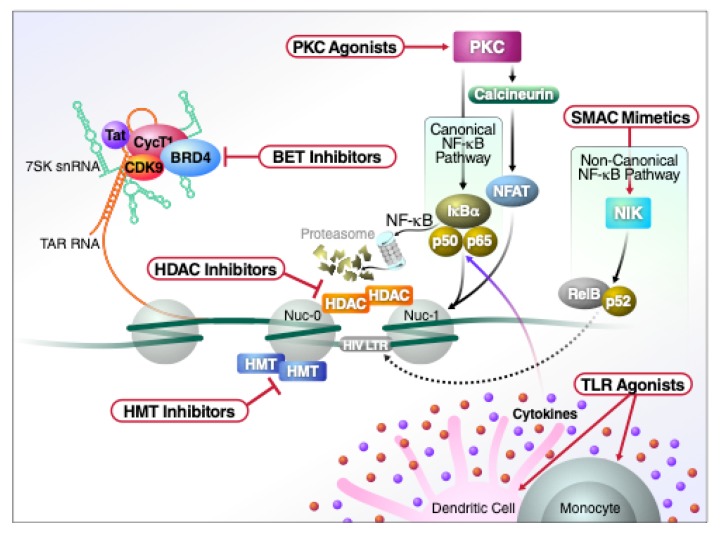
Schematic representation of the major classes of LRAs and their molecular mechanism of action.

To eliminate latently infected cells, one approach is to activate expression of the latent virus under the cover of ART. These virus-expressing reservoir cells then either die due to (i) a viral cytopathic effect, as occurs in primary infection, or (ii) immune cell clearance triggered by their acquisition of “immunological visibility”. Key to implementing the “reduce and control” strategy is the availability of safe and effective latency-reversing agents (LRAs) to accelerate reservoir reduction. Unfortunately, most of the current crop of LRAs are plagued with problems of potency and/or toxicity [69]. Further, these agents only activate a small fraction of the responsive reservoir cells [70], and necessitate repeated administration. The field urgently needs to identify LRAs with greatly improved properties. In the following section, we review the major classes of LRAs (Figure 2) that have been explored in pre-clinical as well as early clinical studies, discussing both their favorable and adverse properties. 

### 2.1. Histone Deacetylase (HDAC) and Histone Methytransferase (HMT) Inhibitors

In latent reservoir cells, HIV transcription is silenced by a combination of epigenetic mechanisms mediated by histone deacetylases and histone methyl-transferases. Inhibitors of these enzymes are capable of activating HIV provirus expression in a variety of cell line models as well as in primary CD4 T cell models of latency, emphasizing a role for epigenetic control of viral latency [71,72,73]. HDAC inhibitors (e.g., valproic acid, vorinostat, and more recently panobinostat and romidepsin) have been tested as single agents in chronically infected individuals on ART [72,74]. However, although spikes in plasma viremia were detectable, no significant changes occurred in the size of the reservoir. Although disappointing, this outcome is perhaps not surprising because: (1) the site of chromosomal integration can affect HIV proviral responses to various agents in vitro, and (2) the more potent HDAC inhibitors directly impair CD8+ T cell and natural killer (NK) effector cell function, reducing clearance of reactivated reservoir cells [75,76]. Of note, vorinostat has been reported to not compromise CTL activity [77]. HMT inhibitors have also been investigated as potential LRAs. Chaetocin, BIX-01294 [78], UNC-0638 [34], AZ391 [79] all reverse latency in cultures of CD4 T cells isolated from HIV positive donors on suppressive ART, but issues of toxicity and potency diminish overall enthusiasm for this class of LRAs [33,80].

### 2.2. BET Inhibitors

The bromo- and extra-terminal domain (BET) inhibitors induce reactivation of latent HIV by inhibiting BRD4, a protein that blocks LTR transcription elongation by preventing the pTEFb complex from binding to Tat [81]. The BET inhibitors also act by selectively binding to and inhibiting the action of the short form of BRD4 (BRD4s) that functions as an HIV corepressor [82]. Specifically, BRD4s directly binds to BRG1, a component of the BAF SWI/SNF chromatin remodeling complex. BET inhibitors negate the repressive effect of this remodeling complex, leading to increases in HIV transcription. The BET inhibitors alone often produce only weak and slowly evolving effects. However, these agents can synergize with PKC activators and they do not compromise cytotoxic effector function [83].

### 2.3. Disulfiram

Disulfiram was identified in a large drug screen as a potential LRA when assayed in a primary CD4 T cell model of HIV latency [84]. Disulfiram is an FDA-approved drug (Antabuse) used in the management of chronic alcoholism. It acts by inhibiting the phosphatase and tensin homolog (PTEN), leading to activation of the Akt pathway and induction of nuclear NF-κB-expression [85]. These effects occur in the absence of broad-scale cytokine production or expression of cellular activation markers. Disappointingly, the promising in vitro effects of disulfiram were not confirmed in vivo. While well tolerated, only modest increases in viral RNA were detected [86,87]. When combined with other LRA classes (e.g., PKC agonists or HDAC inhibitors), no synergy was observed [83]. 

### 2.4. PKC Agonists

Activation of protein kinase C (PKC) initially attracted considerable attention as a mechanism to reverse HIV latency in vitro. PKC agonists, such as prostratin, bryostatin-1 or various ingenol compounds appear among the most potent LRAs, especially when combined with HDAC inhibitors [83,88]. In terms of their effects on HIV latency, bryostatin-1 and prostratin are the best characterized compounds in this group. However, because of their intrinsic toxicity, clinical investigations of bryostatin-1 and other PKC agonists have been slow to progress. When tested in vivo, bryostatin did not obviously alter the transcription of latent HIV at the single low dose tested [89]. As noted, required doses of the PKC activator can be reduced when combined with various synergizing agents including panobinostat, romidepsin, and vorinostat [90]. Such combination strategies could be tested in vivo but their overall toxicity combined with the effects on cytotoxic effector cells and the small fraction of cells activated by each dose dampens overall enthusiasm. 

Ingenols are compounds isolated from the *Euphorbia* family of plants [70]. Ingenol-3,20-dibenzoate exhibits anti-leukemic properties in vitro [91]. Chemically engineered ingenols exhibit latency-reversing activity [92]. For example, Ingenol-3-mebutate, now approved by the FDA as a topical therapy for actinic keratosis, reactivates latent HIV at nanomolar concentrations with minimal CD4 T cell activation/toxicity or release of IFNγ [93,94]. Another ingenol, Ingenol B has been used in combination with the HDAC inhibitor vorinostat to treat SIV-infected pigtail macaques previously suppressed with ART for 400 days. SIV viral load increases were observed in both plasma and the CSF with distinct viruses emanating from these two compartments [95]. Of note, it is unclear whether “shock and kill” approaches can be deployed to attack virus residing in the human CNS reservoir. This approach might simply be too toxic for the neurons intertwined with microglia harboring latent virus. Of note, these LRAs also alter properties of the blood-brain barrier increasing its permeability and allowing trafficking of proinflammatory cells that might paradoxically propel viral seeding of the CNS [96]. 

Ingenol-3-angelate (also known as PEP005) is yet another member of this family approved for the treatment of actinic keratoses [94]. Ingenol-3-angelate also reactivates latent HIV through the induction of NF-κB both alone and in a modestly synergistic manner with JQ1 in vitro [97]. Other ingenol compounds, like extracts from *Euphorbia kansui*, are currently being tested in human clinical trials for HIV latency-reversing activity (see NCT02531295). Kansui is an herbal supplement that has been prescribed for thousands of years in traditional Chinese medicine, often consumed as a tea. The first clinical trial of kansui as a latency-reversing agent in HIV-infected individuals was projected to complete in December 2020 but no results have yet been reported.

### 2.5. Toll-like Receptor (TLR) Agonists

TLRs correspond to membrane receptors that recognize conserved pathogen-associated molecular patterns (PAMPs) comprised of lipids, proteins, RNA/DNA or carbohydrates present in various bacterial, viral, fungal or protozoan pathogens [98]. These TLRs play a key role in initiation of the innate immune response. After binding to their ligands, the TLRs commonly induce activation of NF-κB, AP1 and various interferon regulatory factors (IRFs). Many TLR agonists are now being evaluated as potential LRAs, with agonists of TLR3, TLR7 and TLR9 already advancing into human trials [99]. MGN1703, a TLR9 agonist, induced HIV plasma RNA in 6 of 15 study participants concomitant with increased activation of NK and CD8 T cells, but no reduction in latent reservoir size was observed [100]. Dual TLR2 and TLR7 agonists such as CL413 have shown potent NF-κB-mediated HIV-1 reactivation that unfortunately is also accompanied by proinflammatory release of TFNα [101]. In rhesus macaques infected with simian immunodeficiency virus (SIV) and in HIV-infected individuals, both on ART, the administration of the TLR7 agonists GS-986 and GS-9620 led to significant increases in plasma SIV and HIV RNA, respectively, consistent with latency reversal [102,103]. These TLR agonists are also being tested in combination with various therapeutic vaccines. For example, the combination of TLR7 agonist with an AD26/MVA-based therapeutic vaccine led to improved control of rebound viremia when antiretroviral therapy was discontinued in SIV-infected rhesus macaques [104]. The combination of agents was significantly better than either of the single agents alone. Similarly, combining a TLR7 agonist and a V3 glycan-directed broadly neutralizing HIV antibody resulted either in no rebound or a delayed rebound in SHIV-infected macaques taken off ART [105].

### 2.6. SMAC Mimetics

Agents mimicking the action of the second mitochondria-derived activator of caspases, termed SMAC mimetics [106], both activate latent virus and cause HIV-infected cells to die. Other LRAs lack these dual properties. The SMAC mimetics principally activate the non-canonical NF-κB pathway. In contrast to classical or canonical NF-κB activation, the non-canonical pathway produces a slower and longer-lasting transcriptional activation often triggered through a subset of tumor necrosis factor receptors (TNFRs). In complex with cIAP2, TRAF2, and TRAF3, cIAP1 naturally degrades NF-κB-inducing kinase (NIK), preventing p100 processing into p52 [107]. SMAC mimetics induce degradation of cIAP2 via ubiquitylation and proteasome-mediated degradation. When cIAP2 is degraded, NIK expression stabilizes, allowing this kinase to phosphorylate and promote proteasomal processing of the *NFKB2* gene product p100, yielding p52. p52 and its associated Rel protein partner, RelB, then rapidly translocate into the nucleus. Beyond cIAP2, the SMAC mimetics also promote degradation of several other survival factors including BIRC2, BIRC5 (survivin), XIAP and cIAP1 [108,109,110].

SMAC mimetics can also lead to activation of the canonical NF-κB pathway. Accumulation of NIK ultimately leads to phosphorylation and degradation of inhibitor of κB kinase (IκBα), which in turn allows nuclear translocation of the prototypical NF-κB heterodimer p55/RelA] [111]. 

Among the SMAC mimetics tested thus far, SBI-0637142 and LCL161 are able to downregulate BIRC2, leading to proviral transcription [111]. Interestingly, the SMAC mimetic SBI-0637142 produces synergistic induction of HIV expression when combined with HDAC inhibitors, and induces apoptosis within latently infected CD4+ T cells where viral replication has been reactivated [112]. Three different SMAC mimetics including birinapant, GDC-0152, and a benzolactam-related compound, BL-V8-310, were shown to induce this selective cell death within HIV-1 infected central memory CD4 T cells [113]. In a related series of studies, in vitro treatment of infected cultures with the pro-apoptotic drug Venetoclax, which blocks Bcl-2 function, promoted the rapid death of productively infected primary T cells in vitro and a reduction of the latent reservoir in vitro following anti-CD3/CD28 stimulation of the cultures [114].

### 2.7. Summary and Conclusions 

Since initial attempts to attack the reservoir using “shock and kill” began nearly ten years ago [71], this approach has proved disappointing for a number of reasons: (1) the initial LRAs tested either lacked potency or exhibited unacceptably high levels of toxicity both in vitro and in vivo [115,116]; (2) after a single dose, the tested LRAs only reactivate a small fraction of cells within the latent reservoir [70,117], indicating that serial administration of the agent will be required, placing toxicity issues front and center; (3) HIV can establish viral reservoirs in the central nervous system (CNS) [118], where certain LRAs may not enter, and “shock and kill” strategies may simply be too toxic for neuronal survival; and (4) CD8 T cells in HIV-infected individuals display markers of cell exhaustion and immune dysfunction that are accentuated by various LRAs, leading to a compromised ability to clear reactivated reservoir cells [119]. Clearly, the current crop of LRAs are not up to the task. An ideal LRA will activate a substantial fraction of the latent reservoir but at the same time exhibit a strong safety profile allowing for repeated dosing without excessive T cell activation or cytokine release. Drug combinations yielding synergistic effects will likely be required especially if latency is multifactorial and different LRAs hit different points of viral repression. The lack of highly active and safe LRAs represents a major deficit in HIV cure research and is undermining our ability to implement the “reduce and control” strategy. Of all of the LRAs thus far studied, SMAC mimetics appear the most promising. Their ability to both activate viral gene expression and to preferentially induce apoptosis in these virus-expressing reservoir cells is unique (a single agent that both “shocks” and “kills”). The question is whether these drugs can be given over a long term. Some dose-limiting toxicities including cytokine release syndrome has been observed in a small subset of treated cancer patients. Other side effects described include fatigue, nausea, vomiting, diarrhea and anorexia [120].

## 3. “Block and Lock”: An Alternative Strategy for A Functional Cure 

Because of the noted limitations with the current collection of LRAs, new approaches for neutralizing latent HIV proviruses must be actively explored. One such strategy, termed “block and lock” is conceptually the converse of “shock and kill”. The goal in “block and lock” is to permanently silence the transcriptional activity of the virus, thereby allowing safe withdrawal of ART. Precedent for such neutralization is found with the human endogenous retroviruses (HERVs) that occupy approximately 7% of the human genome [121]. Although some of these HERVs are rendered non-functional by crippling mutations within their open-reading frames, many are silenced through histone trimethylation, DNA methylation and RNAi -dependent pathways [122]. Importantly, it might be possible to deploy these same mechanisms to permanently silence integrated HIV proviruses, thereby neutralizing the latent reservoir. 

Such “block and lock” approaches have conceptual advantages over “shock and kill”. The latter carries the risk of reservoir expansion if kill mechanisms are not effective, and in particular if ART concentration is inadequate in tissues where viral reactivation is occurring [123]. “Block and lock” avoids these complications. Complete transcriptional silencing of the virus would also eliminate the low-level virus production that occurs during ART when virus is intermittently expressed in reservoir cells [124]. These small amounts of virus may drive low level chronic inflammation and immune activation. Even partial silencing could allow reduction in the effective size of the reservoir so that an engineered immune response might be able to keep the residual virus in check. Alternatively, “shock and kill” might be used to initially reduce the size of the reservoir, and then “block and lock” deployed to control the small residual reservoir, perhaps as an adjunct to an engineered immune response. 

**Figure 3:**
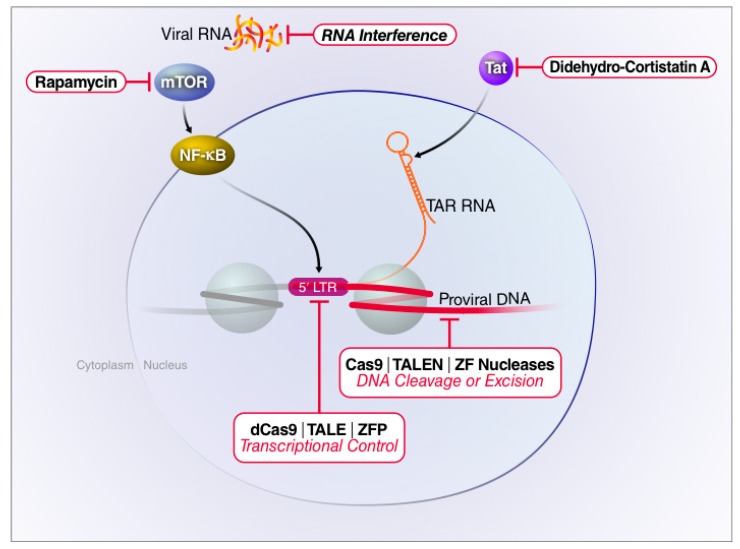
Targeting of host or viral factors to silence latent HIV proviruses. Therapeutics devised to inhibit HIV-1 replication may target different stages of the viral life cycle. The small molecule drug rapamycin blocks viral transcription initiation by inhibiting mTOR and AKT-mediated induction of NF-κB. Didehydro-cortistatin A interferes with Tat-mediated transactivation by blocking Tat binding to TAR, thereby reducing RNA Pol II elongation. RNA interference predominantly acts by degrading viral transcripts. Gene editing technologies directly target proviral DNA by either excising or corrupting the viral genome. CRISPR interference (CRISPRi) does not cleave the proviral DNA. Rather, a catalytically inactive Cas9 (dCas9) protein is fused to a Krüppel associated box (KRAB) domain and directed to the HIV-1 LTR by sgRNAs. This fusion protein recruits transcriptional repressors producing epigenetic changes associated with gene silencing.

Mechanistically, “block and lock” could target either the viral or the host factors involved in HIV replication, transcription or translation (Figure 3). Although attractive due to reduced resistance, targeting of host proteins has the potential to affect multiple signaling pathways leading to unwanted and potentially unpredictable side effects. Virus-directed strategies could be aimed not only at the integrated proviral DNA but also at viral RNAs or even viral proteins. In recent years, various approaches have been proposed and tested, some in simplified and non-physiological model systems but others in vivo, including in human clinical studies. We will summarize the most promising approaches in the following sections. 

### 3.1. Pharmacologic Inhibition of Host Factors

One example of a small-molecule drug qualifying as a “block and lock” or latency-promoting agent (LPA) is the macrolide rapamycin (sirolimus). Rapamycin is the eponymous inhibitor of the mammalian target of rapamycin (mTOR), a protein complex that controls a wide range of cellular activities including differentiation, viability and cell growth [125]. In the context of HIV, rapamycin downregulates surface expression of the CCR5 [126] and CXCR4 [127] co-receptors, thus inhibiting R5- and X4-tropic viral entry. Rapamycin also interferes with viral transcription [128] and latency reversal [129]. As of August 2019, two clinical trials with rapamycin have been conducted examining drug impact on HIV persistence and immune activation/inflammation. One study involved a combination of rapamycin with the CCR5 agonist Maraviroc (NCT02990312), while the second tested rapamycin alone (NCT02440789). Both studies are now complete, but results are not yet available. Of note, rapamycin alone is unlikely to allow the discontinuation of ART, but it could reduce viral transcriptional activity and reservoir size [130]. Rapamycin might also be used in combination with various LRAs to block LRA-mediated induction of a cytokine storm [131]. However, as for many other host-directed therapies, it is likely that the immunosuppressive effects of this drug will preclude its long-term use.

HIV transcription is critically dependent on the regulatory action of the viral trans-activator protein (Tat) [132]. The cellular cyclin-dependent kinase 9 (CDK9) is a key host factor that is required for Tat-dependent viral gene expression. CDK9 assembles with cyclin T1 to form the PTEF-b complex that binds to Tat and is recruited to HIV’s TAR element by the RNA binding properties of Tat. PTEF-b phosphorylates the C-terminal domain of paused RNA Pol II polymerase complexes bound to the HIV proviral template, thus promoting transcriptional elongation by the complex [132]. Not surprisingly, inhibitors of CDK9 impair HIV-1 activation in both cell line models of HIV-1 latency and in infected peripheral blood lymphocytes analyzed in vitro [133,134,135,136,137]. The CDK9 antagonist, indirubin 3’-monoxime, induces a sustained reduction of viremia in the absence of significant cytotoxicity or other severe adverse effects in a humanized mouse model of chronic HIV infection [138]. However, the ability of CDK9 to pair with different cyclins to affect RNA Pol II initiation, elongation, and termination predicts that long-term targeting of CDK9 will be associated with unacceptable adverse effects. 

Vargas et al. [53] recently took a more unbiased approach in an attempt to identify antagonists of HIV-1 expression, screening a large library of clinically applicable kinase inhibitors in a cell line model of viral latency. As expected, this screen identified protein kinase C inhibitors but surprisingly also danusertib, an Aurora kinase inhibitor, and PF-3758309, a PAK4 (p21-activated kinase 4) inhibitor, as agents that block latency reversal in cell lines as well as CD4+ T cells from HIV-1 infected individuals on long-term ART. The precise mechanism of action of these different kinase inhibitors remains unclear and again, overall enthusiasm for their use is tempered by the central role that these kinases play in cell mitosis, adhesion, migration, proliferation, and survival. Other previously identified latency-promoting agents include curaxin (CBL0100), a small molecule targeting the chromatin transcription complex (FACT) that efficiently blocks HIV-1 transcription in vitro and ex vivo [139]; levosimendan, which blocks viral replication through inhibition of the phosphoinositide 3-kinase pathway [140]; and ABX464, a drug that interacts with the Cap Binding Complex (CBC) and inhibits the Rev-dependent nuclear export of unspliced HIV transcripts [141]. ABX464 not only suppresses viral replication in vitro, but also delays viral rebound in humanized mice [141] and reduced viral reservoir size in early clinical studies [139,142]. Again however, the question remains whether the beneficial antiviral effects of these drugs outweigh the potentially harmful consequences of a long-lasting and systemic suppression of the respective cellular pathways.

Recently, lens-epithelium-derived growth factor p75 (LEDGF/p75) emerged as a key host determinant of HIV-1 latency, which, by controlling viral integration site selection [143,144], impacts the transcriptional activity of the provirus. LEDGINs, small molecules that block interactions between the viral integrase and LEDGF/p75 [145], retarget proviral integration to sites associated with a deeper state of latency [146]. Since LEDGINs also suppress viral spread by interfering with viral particle assembly [147], these agents could be used to both impair viral spread and, if such spread occurs, to promote latent proviruses that are more resistant to reactivation. However, previously established latent reservoirs are not predicted to be affected by LEDGINs, undermining their utility as curative agents [147].

### 3.2. Small Molecule Inhibition of HIV Tat 

The HIV Tat protein is a major determinant of HIV replication and persistence, boosting viral gene expression by at least two orders of magnitude [148]. Weinberger and colleagues suggest that the rare stochastic loss of Tat expression drives the induction of latency, independent of the state of activation of the host cell [149,150,151]. As such, inhibition of Tat function might lead to marked silencing of the virus (reviewed in [152,153]). 

Didehydro-cortistatin A (dCA) has been identified as a Tat inhibitor. dCA is an analogue of a steroidal alkaloid found in the marine sponge *Corticium simplex.* dCA binding to Tat impairs its interaction with TAR, thus interrupting Tat-mediated trans-activation [154]. Dr. Susana Valente and her colleagues have demonstrated that dCA efficiently disrupts the Tat-induced transcriptional feedback loop and establishes a long-term suppression of HIV transcription that persists even after removal of dCA. These long-lasting effects appear to involve heterochromatin formation within the HIV-1 LTR [155,156,157]. When tested in the BLT humanized mouse model, dCA significantly reduces viral mRNA levels in tissue reservoirs and delays viral rebound following treatment interruption [157]. Viral mutations conferring resistance to dCA have also been identified and are associated with heightened basal HIV-transcription leading to increased viral cytopathic effect and enhanced immune-mediated clearance [158]. It will be interesting to assess how long after drug removal the silencing effects of dCA persist, and whether this drug can be partnered with other agents to achieve synergistic silencing of latent HIV proviruses. 

### 3.3. Targeting HIV-1 mRNAs

Since the advent of RNA interference (RNAi) in the 1990s (reviewed in [159,160]), this technology has rapidly expanded into the HIV field [161]. As compared to pharmacological approaches, RNAi has the intrinsic advantage of its sequence specificity. However, this same strength makes RNAi subject to viral evolution and emergence of resistance [162,163,164]. To circumvent viral evasion, multiple, highly conserved regions can be targeted where mutations result in a severe fitness cost [165,166,167]. In general, siRNAs work quite well in in vitro systems [161,167], but in vivo performance is often disappointing due to problems with delivery and the necessity for repeated administration to sustain effective siRNA levels (reviewed in [165,168,169]). Nevertheless, some success has been reported in humanized mouse models where interfering RNAs are delivered by lentiviruses, nanoparticles or dendrimers and aptamers (reviewed in [170]). Early human clinical trials have been limited, for ethical reasons, to HIV-1 infected individuals with concurrent cancers. DiGiusto and colleagues transduced hematopoietic stem and progenitor cells (HSPCs) ex vivo with RNA-based anti-HIV moieties and successfully engrafted these cells back into patients after radio- or chemotherapy [171]. Later studies utilized autologous hematopoietic cells, transduced with shRNA targeting CCR5 and the HIV-1 LTR. These cells were returned to the otherwise healthy HIV-1 infected donors (reviewed in [170]). However, in the initial trial, inconclusive results were obtained due to low in vivo frequencies of modified cells [171] while in the other case, the trial results remain pending (see [170] for trial identifier). 

RNAi has often been proposed as a complement to current antiretroviral regimens. However, an often-underestimated aspect of RNAi-based approaches is its predilection to produce off-target effects [172], both sequence-specific and sequence-independent [173]. As such, long-term RNAi administration of siRNAs could be problematic. 

## 4. Gene Editing Strategies to Attack Latent HIV Proviruses

**Figure 4:**
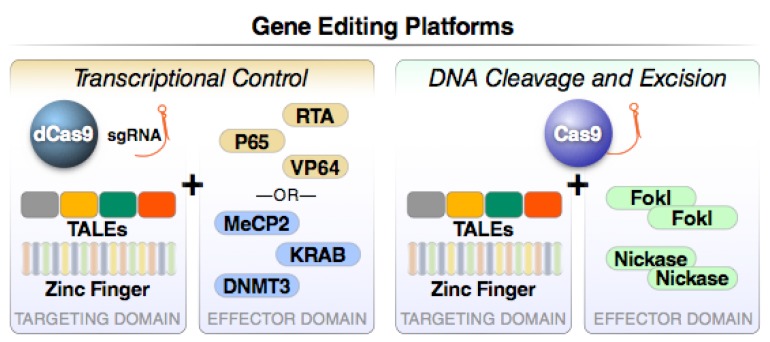
Major gene editing platforms operating through transcriptional control or DNA cleavage and excision. Two functions are present in all gene editing platforms. The first component directs sequence-specific DNA binding while the second promotes either frank DNA cleavage or the recruitment of epigenetic modifiers that positively or negatively control target gene transcription. For example, a catalytically inactive or dead version of Cas9 (dCas9) can be fused to a strong transcriptional activation domain from the RelA transcription factor (NF-κB p65), the replication and transcription activator (RTA), or repeats of the HSV VP16 protein (VP64), to induce transcriptional activation. Alternatively, transcriptional inhibition can be induced by fusing dCas9 to Krueppel-associated box (KRAB) proteins, methyl CpG binding protein 2 (MeCP2), or DNA methyl transferase 3A (DNMT3a). These “baits” effectively recruit transcriptional repressors that silence gene activity via epigenetic mechanisms.

All previously described “block and lock” approaches require at least minimal viral transcription and/or translation to be effective. However, recent studies suggest that viral reservoirs are mostly devoid of significant virus replication [174] and HIV gene expression [175,176]. Thus, most latently infected cells would not be affected by treatments targeting either viral transcripts or viral proteins, except during the narrow window of viral reactivation. Therapeutic targeting of the proviral DNA overcomes these limitations. A key prerequisite for this approach is a highly specific and easily adaptable DNA-binding moiety. In recent years, different DNA-binding proteins have been explored, including transcription activator-like effectors (TALEs), Zinc finger proteins (ZFPs), homing endonucleases (HEs), and clustered regularly interspaced palindromic repeats (CRISPR)-associated nucleases like Cas9 [177,178,179] (Figure 4). In their natural context, most DNA binding proteins also exert effector activities. For example, both HEs and Cas9 cleave double stranded DNA, TALEs activate gene expression and ZFPs exert diverse functions including activation and inhibition of gene expression. For tailored applications, these proteins are often fused with specific effector protein domains including endonucleases, transcriptional activators, or transcriptional repressors. The transcriptional regulators act directly while the endonucleases act more indirectly by inducing double-strand breaks (DSBs) in their target sequences. Cellular mechanisms repair these DSBs, either via homology-directed repair/recombination (HDR) producing a clean, “scarless” repair, or, more frequently, via non-homologous end joining repair (NHEJ) that conversely generates a genetic scar characterized by insertions or deletions (indels). Indels within open reading frames have a two in three chance of producing frame-shift mutations that will disrupt natural viral protein production. In the following sections, we review how these gene editing approaches are being used to neutralize HIV. 

### 4.1. Homing Endonucleases

Homing endonucleases are naturally occurring DNA-cleaving enzymes that confer mobility to their own open reading frames [180]. They are highly specific for relatively long (12–40 bp) pre-defined DNA sequences, a property that unfortunately limits their utility. Only one report has appeared using HEs to target HIV. This study revealed only a modest reduction of viral gene expression in a cell line model of viral latency [172]. It seems quite unlikely that the HEs will gain traction as an effective anti-HIV therapeutic. 

### 4.2. Zinc Finger Proteins

Zinc fingers are among the most abundant DNA-binding motifs in eukaryotes [181]. Natural zinc finger proteins (ZFPs) typically require at least three fingers to achieve sequence-specific binding [181]. Universal libraries and specific recognition codes are now available [182], enabling a modular assembly of sequence-specific ZFPs [183]. However, ZFP targets are limited by sequence requirements, and the engineering of these DNA-binding elements remains cumbersome and time-consuming [184]. A number of studies have utilized engineered ZFPs to target HIV proviruses. For example, ZFPs have been fused with nucleases [185,186,187] and transcriptional repressors [188,189,190,191,192] or activators [193], and tested in HIV-1 reporter cell lines and cell line models of HIV-1 latency, as well as primary human peripheral blood mononuclear cells [185,188,190]. 

ZFPs have also been used to target the CCR5 co-receptor for HIV. This strategy seeks to phenocopy a naturally occurring homozygous deletion in the CCR5 gene (CCR5 Δ32/Δ32) known to confer high-level resistance to R5-tropic HIV-1 infection [194,195]. Mutation of this gene limited HIV-1 infection and spread in humanized mouse models [196,197]. CCR5-targeting ZFPs were also tested in a small-scale clinical study where autologous CD4 T cells from HIV-infected individual were edited ex vivo and reinfused. The CCR5-modified cells survived longer than the unmodified T cells, and plasma viral loads were lower during ART interruption [198]. Other clinical studies targeting CCR5 are currently recruiting or were recently completed but have not yet been reported (summarized in [199]). These studies generally support the feasibility of ZFP-based gene editing approaches to attack HIV via deletion of its key co-receptor. In contrast, targeting the latent HIV provirus for direct cleavage may be more difficult because these cells are rare and sequence variability may be encountered. In contrast, editing the CCR5 receptor in hematopoietic stem cells represents an exciting possibility for creating immune cells that are intrinsically resistant to HIV infection. 

### 4.3. TALEs 

Transcription activator-like effectors (TALEs) are DNA binding proteins first identified in *Xanthomonas* bacteria infecting plants [194]. TALEs bind DNA via arrays of highly conserved 33–35 amino acid repeats. A single TALE repeat within an array binds specifically to individual DNA bases due to two hypervariable residues present at position 12 and 13 within the repeat [194]. The protein-DNA code of TALE repeats has been deciphered and enables the rapid and modular design of highly specific TALE-DNA binding proteins for virtually any given target sequence [200]. The majority of HIV-1 related studies have focused on transcription activator-like effectors nucleases (TALENs)––fusion proteins composed of specific TALE arrays and the FokI endonuclease (reviewed in [194]). In two separate studies, the introduction of TALENs targeting HIV proviral sequences within the LTR region in infected cell lines resulted in robust reduction of viral replication [201,202] and the excision of proviral DNA [201]. 

TALENs are also showing promise in other strategies. Like ZFPs, TALENs have been used to knock out the CCR5 gene in cell culture [203,204,205,206], permitting efficient gene editing and protection of cells from HIV infection [207] with minimal off-target activity and low cytotoxicity. TALE-activators, corresponding to TALEs fused to potent transcription activators, have been developed and shown to effectively reactivate latent HIV-1 in cell line models [208,209,210] and in PBMCs from ART-suppressed individuals [210]. 

TALE technology combines several features that are quite favorable for gene editing applications. TALEs are specific, easy to design and generally less cytotoxic than other DNA targeting proteins [206]. On the downside, TALEs are relatively large and contain repetitive sequences that can complicate cloning [211] and interfere with incorporation into gene delivery vectors [212,213]. 

### 4.4. CRISPR

The recent discovery of clustered regularly interspaced short palindromic repeats or CRISPR has revolutionized gene editing in mammalian cells. CRISPR repetitive sequences were initially discovered in bacteria [214], where they form part of a larger protective response aimed at bacteriophages and other intrusive DNAs [215,216]. Bacterial helicases such as Cas9 bind CRISPR RNAs (crRNA), which then target pathogenic DNA leading to Cas9-mediated cleavage and degradation of the external threat [216]. The ability of CRISPR to direct double-strand DNA cleavage by Cas9 at specific sites through the action of crRNAs (or small guide RNAs, sgRNAs) has created an immensely powerful gene editing tool. CRISPR/Cas9 applications are also not limited to genome editing by DNA cleavage. The introduction of a mutation within the two nuclease domains, RuvC and HNH, results in a catalytically inactive protein termed “dead” Cas9 (dCas9). dCas9 can be fused to an activator or conversely to a host protein that effectively recruits repressor complexes to achieve either gene activation or gene silencing [217]. One challenge with this approach is the fact that Cas9 and dCas9 are bacterial proteins and thus highly immunogenic [218]. This constraint will likely limit serial dosing of cocktails containing Cas9/dCas9.

CRISPR/Cas9 technology was first applied to HIV-1 in 2013 [219], with several rapidly ensuing studies (reviewed in [216]). Proviral cleavage by CRISPR was often tested in cell lines, like HEK 293T cells and HeLa [219,220,221,222,223], which unfortunately do not resemble natural targets of HIV. However, this technology was also shown to be effective in more relevant T cell lines [219,220,221,222,223] and in cell line models of HIV-1 latency [220,222,223,224]. Importantly, CRISPR/Cas9 has now been used to successfully edit HIV-1 in in vitro-infected primary cells and in PBMCs from ART-suppressed individuals [225]. Multiple studies have also shown the successful application of CRISPR-Cas9 in HIV-1 infected humanized mice [226,227,228,229]. These developments culminated in an interesting humanized mouse study showing that the combination CRISPR/Cas9 and a long-acting slow-effective release ART (LASER ART) achieved apparently complete viral clearance in the absence of any detectable Cas9-mediated off-target effects [229]. 

One concern with CRISPR approaches directed against HIV-1 is that the double strand breaks and resulting mutations, while crippling the majority of the edited viruses, may in some cases facilitate viral escape [230,231,232,233]: some of the NHEJ-induced random mutations may be silent and not reduce viral fitness. Such mutant viruses would no longer be susceptible to further CRISPR suppression and could rapidly repopulate the target cell pool [230,231,232]. In vitro, multiplexing with several guide sequences and targeting multiple highly conserved viral regions could minimize this problem [224,234]. A larger issue is the frequency of off-target editing by the CRISPR/Cas9 system [235,236].

Several prior studies have also focused on knocking out the HIV co-receptors, CCR5 and CXCR4, as a means to block viral spread. As expected, CRISPR-Cas9-mediated knockout of the CCR5 gene inhibited HIV-1 infection in induced pluripotent stem cell-derived monocyte/macrophages [237], primary CD4+ T cells [238], and in vivo [239,240]. Similarly, CXCR4 knockout was shown to confer resistance to X4-tropic virus infection [241,242,243]. However, in view of the importance of CXCR4 in the development of hematopoietic cells [244], the clinical feasibility of this latter knockout approach is more problematic. 

A number of CRISPR-based approaches have made use of the catalytically inactive dCas9 mutant. Fused with a potent transcriptional activator, dCas9 enables specific induction of gene expression, an approach termed CRISPR activation (CRISPRa) [217]. This approach supports strong in vitro re-activation of latent HIV-1 [245,246,247,248,249,250,251] at levels greater than most tested latency-reversing agents [246]. In some cases, viral burst size is increased sufficiently to induce viral cytopathic effects [250]. CRISPRa has been successfully tested in cell lines and cell line models of latency [245,246,247,248,249,250,251], but not yet in primary cells or in vivo. CRISPRa could also be used to upregulate protective, antiviral factors in order to control HIV-1 infections. In early proof-of-concept studies, CRISPRa has been shown to activate the expression of the HIV-1 restriction factors APOBEC3 [252] and BST-2/Tetherin [253] and to inhibit HIV-1 infectivity.

Conversely, catalytically inactive or “dead” Cas9 (dCas9) can be used to suppress viral transcription via CRISPR interference, or CRISPRi [246]. A preliminary study suggests that suppression can be significantly improved by fusing dCas9 to the Krueppel-associated box domain (KRAB) that in turn recruits other repressor complexes [221]. More focused studies are necessary to thoroughly explore the efficacy and applicability of the CRISRPi/CRISPRa modalities in HIV-1 cure approaches. Drawbacks of this approach include issues of sustained gene delivery and the intrinsic immunogenicity of the bacterial Cas9 protein.

### 4.5. Summary and Conclusions

Each of the described gene editing approaches exhibits advantages and disadvantages. Strengths and weaknesses vary depending on the target cell population and the editing context. For example, the problematic immunogenicity of the bacterial Cas9 protein that occurs in vivo is of less concern when “hit and run editing” is performed ex vivo and modified cells then reinfused. The size of the editing components also varies (CRISPR >4000 base pairs vs. ZFP <1000 base pairs), which can limit the use of select transfer vectors. A summary of all of the genome editing tools discussed in this review is provided in Table 1. Importantly, only a few studies have performed actual side-by-side comparisons of these different editing systems. The purpose of the table is to provide a global overview of the features of HE, ZFP, TALE and CRISPR platforms based on published reports and reviews. 

### 4.6. Challenges 

The majority of the anti-HIV gene therapy approaches directly attack either the virus itself or host genes that are critical for viral growth and spread. As described above, nuclease-based strategies result in double strand breaks (DSBs), which are predominantly repaired by error-prone non-homologous end joining. However, DSBs can also produce toxic effects [263], potentially promoting neoplastic transformation or other life-threatening disorders [264]. In the case of HIV-infected individuals who are doing well on ART, therapeutic approaches involving the introduction of DSBs may be judged too risky. Conversely, the use of inactive Cas9 fused to transcriptional activators or inhibitors could be deployed with less toxicity, but the intrinsic immunogenicity of the bacterial Cas9 protein complicates repeated use. Other major issues relate to gene delivery and sustained expression of the gene cargo. In the case of HIV, the ultimate approaches must be safe, effective and scalable, including deployment within the developing world where HIV is hitting the hardest. 

## 5. Concluding Thoughts

Simply put, the HIV cure field has entered a period of considerable uncertainty. Initial enthusiasm for “shock and kill” as a cure strategy has receded chiefly because safe and effective LRAs have been difficult to identify. Disturbingly, many of the most promising shocking agents actually undermine CTL effector function. Further, in vitro activations studies revealed that only a small percentage of the replication-competent HIV reservoir is activated with strong LRAs [8,68,117]. As such, multiple rounds of LRA treatment will be required and the toxicity profile of these agents is critical. Clearly, the majority of the current LRAs are not up to the task, and further basic research is necessary to better understand why previous cure approaches failed and how future attempts may overcome existing roadblocks. 

**Figure 5:**
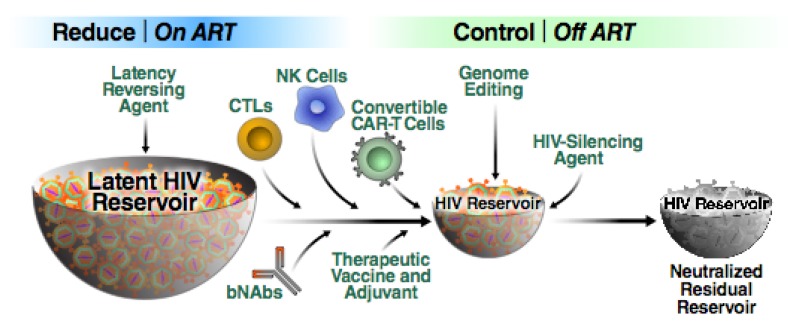
Schematic representation of the “reduce and control” approach. First, a combination of effective, non-toxic LRAs are employed in order to render reservoir cells visible to the immune system. The reservoir is then reduced in size likely through the use of multiple agents including bNAbs, various killer cells and therapeutic vaccines. The residual virus can be controlled by the same agents used to reduce the size of the reservoir. However, this shrunken reservoir could form an attractive target for additional transcriptional silencing approaches. We believe it likely that some form of this combinatorial “reduce and control” strategy will emerge as a means of achieving a sustained HIV remission in the absence of ART.

Ultimately, if sufficient latency reversal is achieved in vivo, a variety of killing mechanisms could be deployed. It is unclear what immune interventions will be the most successful but bNAbs, therapeutic vaccines, NK cells coupled with bNAbs for ADCC, CTLs, and even engineered killer cells should be further explored (Figure 5). These agents could be used to both initially reduce the size of the reservoir and then control the small residual pool of viruses. 

Within this review, we have not addressed pharmacological killing agents that augment elimination of virus-infected cells, which have been the subject of a recent review by Kim et al. [115]. Potentially, one or more of these killing compounds may be included in “shock and kill” cocktails. At the moment, the SMAC mimetics, which both reverse latency and promote the death of reactivated reservoir cells, are the most interesting LRAs and merit careful examination in vivo. Analytical treatment interrupt (ATI) trials remain the only definitive way to assess if cure therapies lead to a sustained ART-free remission. In a recent study by Clarridge et al. [265], it was shown that ATI did not significantly increase the size of the reservoir, nor did it inflict permanent damage to the immune system. Although cumbersome, ATI is likely to remain the gold standard for definitive testing of promising cure interventions. 

In our opinion, “shock and kill” as a stand-alone approach to an HIV cure is unlikely to be effective. In fact, in view of the stochastic nature of latency reversal [8] and the fractional induction of virus observed, a more robust approach than offered by “shock and kill” is needed. Analogous to the success of combination antiretroviral therapy attacking different steps in the HIV life cycle, we believe a combinatorial approach will be required to neutralize the viral reservoir. We predict the first cures will not involve complete viral eradication but rather “functional cures” where the size of the reservoir is reduced and the residual virus controlled by an engineered immune intervention as described above. We further suggest that “block and lock” or transcriptional silencing approaches will likely play an important role in the control of residual viremia after the reservoir has been reduced in size. In this context, the emergence of Tat inhibitors like didehydro-cortistatin A, which exhibit virus specificity and sustained viral suppression even after drug withdrawal, is intriguing and certainly worthy of detailed investigation. The sequence-specific nature of gene editing platforms is also attractive for attacking HIV. The flexibility and modularity of the RNA guided CRISPR system is an undeniable advantage that seems to outweigh some of its inherent shortcomings. However, safety concerns have to be thoroughly addressed before gene therapies can be administered widely to HIV-infected individuals, especially individuals who are doing well on ART. An absolute key will be getting a “lock” into “block and lock” that allows for permanent silencing of HIV proviruses in the absence of continuous drug treatment. Simply replacing ART with another set of long-term drugs would not be a desirable outcome. On a global scale, an ability to reproducibly achieve a sustained remission of HIV in the absence of ART in resource-limited environments would not only save billions of dollars annually spent on daily ART, but would also transform sub-Saharan’s Africa’s ability to end it longstanding struggle against HIV/AIDS. 

## Figures and Tables

**Table 1:** Properties of common gene editing platforms.

	HE	ZFP	TALE	CRISPR
**Target sequence constraints**	High (pre-defined targets)	No constraints (any sequence)	No constraints (any sequence)	Low (PAM required)
**Target size** [213,254]	12–44	18–36	24–40	17–23
**Design and assembly** [213]	Difficult	Moderate	Easy	Very easy
**Toxicity** [206,211,255]	Low	High	Low	Unclear, high in some cell lineages
**Specificity** [211,254,256,257]	High	Low to Moderate	Moderate to High	Low to Moderate
**Multiplexing suitability** [254]	Low	Low	Moderate	High
**Source** [254]	Organelles, Bacteria, Phages	Bacteria, Eukaryotes	Bacteria	Bacteria
**Cost to generate knockout reagent** [254]	4000–5000 USD	5000–10,000 USD	<1000 USD	<100 USD
**Immunogenicity** [218,254]	Unknown	Low	Unknown	Prevalent pre-existing immunity
**Size of effector protein in kDa** [258,259]	<40	~40	~105	~160
**Average length of effector protein** [258,260]	200–300 aa	120–180 aa	660–700 aa	1400 aa
**Sensitivity to chromatin condensation** [261,262]	Sensitive to chromatin compactation and CpG Methylation	Binds condensed and hypermethylated DNA	Potentially decreased binding of condensed DNA	Targeting of hypermethylated CpG islands may be limited
